# Immune-Based Therapies and the Role of Microsatellite Instability in Pancreatic Cancer

**DOI:** 10.3390/genes12010033

**Published:** 2020-12-29

**Authors:** Michele Ghidini, Andrea Lampis, Milko B. Mirchev, Ali Fuat Okuducu, Margherita Ratti, Nicola Valeri, Jens C. Hahne

**Affiliations:** 1 Division of Medical Oncology, Fondazione IRCCS Ca’ Granda Ospedale Maggiore Policlinico, 20122 Milan, Italy; michele.ghidini@policlinico.mi.it; 2 Division of Molecular Pathology, The Institute of Cancer Research, London SM25NG, UK; andrea.lampis@icr.ac.uk (A.L.); mratti.cremona@gmail.com (M.R.); nicola.valeri@icr.ac.uk (N.V.); 3 Centre for Evolution and Cancer, The Institute of Cancer Research, London SM25NG, UK; 4 Clinic of Gastroenterology, Medical University, 9002 Varna, Bulgaria; mbmirchev@yahoo.com; 5 Pathologie Länggasse, 3063 Bern, Switzerland; okuducu@patholaenggasse.ch; 6 Medical Department, Division of Oncology, ASST di Cremona, Ospedale di Cremona, 26100 Cremona, Italy; 7 Department of Medicine, The Royal Marsden NHS Foundation Trust, London SM25NG, UK

**Keywords:** pancreatic cancer, microsatellite instability, Lynch syndrome, mismatch repair system, immune-based therapy

## Abstract

Pancreatic cancer is one of the most aggressive malignancies with limited treatment options thus resulting in high morbidity and mortality. Among all cancers, with a five-year survival rates of only 2–9%, pancreatic cancer holds the worst prognostic outcome for patients. To improve the overall survival, an earlier diagnosis and stratification of cancer patients for personalized treatment options are urgent needs. A minority of pancreatic cancers belong to the spectrum of Lynch syndrome-associated cancers and are characterized by microsatellite instability (MSI). MSI is a consequence of defective mismatch repair protein functions and it has been well characterized in other gastrointestinal tumors such as colorectal and gastric cancer. In the latter, high levels of MSI are linked to a better prognosis and to an increased benefit to immune-based therapies. Therefore, the same therapies could offer an opportunity of treatment for pancreatic cancer patients with MSI. In this review, we summarize the current knowledge about immune-based therapies and MSI in pancreatic cancer.

## 1. Introduction

One of the most lethal malignant neoplasms worldwide is pancreatic cancer with 430,000 deaths and 460,000 new cases in 2018 [1]. Developed countries have the highest incidence and mortality rate of pancreatic cancer [2]. During recent decades pancreatic cancer incidence has increased, and it is likely to increase further during the coming years as the population ages [2,3]. Beside age, significant risk factors for pancreatic cancer are heavy drinking, smoking, obesity diabetes mellitus, dietary factors, physical inactivity and non-O blood group as well as positive family history and genetics (e.g., Lynch syndrome, familial adenomatous polyposis, familial atypical multiple mole melanoma syndrome, Peutz–Jeghers syndrome, Li Fraumeni syndrome, hereditary breast and ovarian cancer syndrome, mutation in genes coding for BRCA2, PRSS1, ATM, K-Ras, STK11, PRSS1/PRSS2, SMAD4, CDKN2A, and p53) [2,4,5,6,7,8,9,10,11,12]. Ethnicity appears to be a risk factor for pancreatic cancer as it has been observed that pancreatic cancer affects more people with African origin compared to other ethnicities [2,3]. It is expected that the number of pancreatic cancer deaths will surpass colorectal cancer in the coming years and become the second leading cause of cancer death in the world [13].

Around 80% of pancreatic cancer cases are caused by sporadically occurring mutations, approximately 10% are related to inherited conditions, and 10–15% have a positive familial history for the disease [14]. Pancreatic cancer can be divided into two main types: (I) the vast majority are adenocarcinoma, and (II) less than 5% of all cases are pancreatic endocrine tumors [15,16].

Since pancreatic cancer is often diagnosed at a late stage, treatment options are limited and traditional chemotherapy is ineffective since the disease has one of the densest stroma of all epithelial tumors that protect cancer cells from treatment [17,18]. Therefore, surgery remains the only curative treatment for pancreatic cancer [19]. Nevertheless, more than 80% of all patients are not eligible for resection at the time of diagnosis [20] and even after curative surgical treatment the five-year survival rate is, at 20%, marginal [21]. Considering all pancreatic cancer patients (including the ones with unresectable tumors) the five-year survival rate is only 2–9% [8,22]. Very often, pancreatic cancer, as with several other cancers, forms distant metastasis [23].

In the adjuvant setting, chemotherapy with 5-fluorouracil, oxaliplatin, and irinotecan (FOLFIRINOX) is the standard of care as this combination treatment significantly prolonged median disease-free survival (DFS) and overall survival (OS) compared to treatment with gemcitabine alone [24]. An alternative treatment is adding capecitabine to gemcitabine and the doublet regimen showed an OS advantage over single-agent gemcitabine [25]. Nevertheless, in case of elderly and unfit patients, chemotherapy with gemcitabine alone is a reasonable choice and it was shown to increase both DFS and OS compared to observation [26]. Borderline and locally advanced disease should be treated with induction treatment to down-stage the mass to attempt secondary surgery. A recent meta-analysis of non-randomized patient cohorts showed that a modified FOLFIRINOX regimen (mFOLFIRINOX) is effective and may be the treatment of choice in this setting of disease [27,28]. In the metastatic setting, both triplet chemotherapy with FOLFIRINOX and combination of gemcitabine and nab-paclitaxel result in better progression-free survival (PFS) and OS rate compared to gemcitabine treatment; therefore, these combination therapies are suitable alternatives for the first-line treatment [24,29,30].

Similarly to other cancer types a low percentage of pancreatic cancers harbor microsatellite instable genotype (MSI-H) and a mismatch repair deficient phenotype (dMMR). In a different series, dMMR pancreatic cancers had a prevalence of 0.8% and 1.3% of total cases [31]. When only intraductal papillary mucinous neoplasm (IPMN)-associated pancreatic cancers were considered, the percentage of cases raised to 6.9% [30]. In one study, it was found that the incidence of MSI-H was comparable in intraductal papillary-mucinous adenoma and intraductal papillary-mucinous carcinoma patients [32]. Of special note, up to now all these studies are based on a limited number of patients [30,32,33,34] and therefore it is not established if MSI is related to a particular type of IPMN. Considering these facts, we will focus in this review on current knowledge of the role of MSI in pancreatic cancer and its potential use for targeted immune-based therapies.

## 2. Lynch Syndrome and Microsatellite Instability (MSI)

MSI is the hallmark of Lynch syndrome and it was first described in colorectal cancer patients in 1913 [35]. Later the definition was broadened and extracolonic tumors have been included [36]. Lynch syndrome patients have either germline mutations in genes coding for DNA mismatch repair genes (like MLH1, MSH2, MSH6, and PMS2) or transcriptional inactivation of these genes. Missing functional DNA mismatch repair proteins results in reduced genome integrity due to missing proof-reading and editing during DNA transcription. Furthermore, variations in microsatellite repetitions occur, thus causing changes in the genome length [37,38,39,40]. This molecular phenotype can be used either as a diagnostic tool by polymerase chain reaction (PCR) amplification of microsatellite sequences or by next-generation sequencing for detection of MSI. Another diagnostic tool is based on immunohistochemistry staining for expression of mismatch repair proteins [38].

In general, Lynch syndrome represents a high risk factor to develop cancer and predisposes to several cancer types including colorectal, endometrial, gastric, ovarian, urinary tract, prostate, small bowel, duodenal, esophageal, hepatocellular, gallbladder, pancreatic cancer, and intrahepatic cholangiocarcinoma [4,36,38,41,42]. Moreover, a defective mismatch repair system results in the accumulation of somatic mutations, leading to higher neo-antigen load, which promotes T-cell activation. Increased neo-antigen expression and recruitment of cytotoxic T-cell can contribute to the increased immunogenicity of cancers with MSI and might enhance the vulnerability of these tumors to immunotherapy [43]. It has been calculated that MSI tumors contain 10 to 100 times more mutations than cancers with an intact DNA mismatch repair system [44].

## 3. MSI and Pancreatic Cancer

The colorectal tumor is the most common tumor among Lynch syndrome families; however, families that carry the MMR (mismatch repair) gene defect have a very high risk to develop pancreatic cancer. Lynch syndrome patients have a nearly 9-fold higher risk of pancreatic cancer in comparison to the general population. Pancreatic tumors caused by Lynch syndrome have often a medullary appearance with prominent lymphocytic infiltration [4,45,46,47]. Pancreatic cancer developed in Lynch syndrome context is normally diagnosed before the age of 60 [4,48] and some can also have different histological subtypes such as intraductal papillary mucinous neoplasms and acinar cell carcinoma [49,50,51]. MSI has been found in several patients’ studies both on resected and metastatic diseases with frequencies between 0% and 75% (Table 1). This wide difference might be related to the patients’ selection criteria and to the different markers used for mismatch repair detection [30,31,49,52,53,54,55,56,57,58,59,60,61,62,63,64,65,66,67,68,69,70,71,72,73,74,75,76]. Nevertheless, the overall rate of MSI pancreatic cancer patients seems to be low (around 2% of all cases) according to studies based on larger series of consecutive pancreatic cancer patients [30,31,77,78]. Most probably, this group needs a special treatment and could benefit from personalized treatment. Considering that 2% to 4% of all diagnosed cancers are mismatch repair-deficient, pancreatic cancer can fit into this range [79,80,81]. In general, microsatellite instability status represents a better prognostic factor for pancreatic cancer patients, potentially derived on a stronger anti-tumor response of the innate immune system [58].

**Figure 1:**
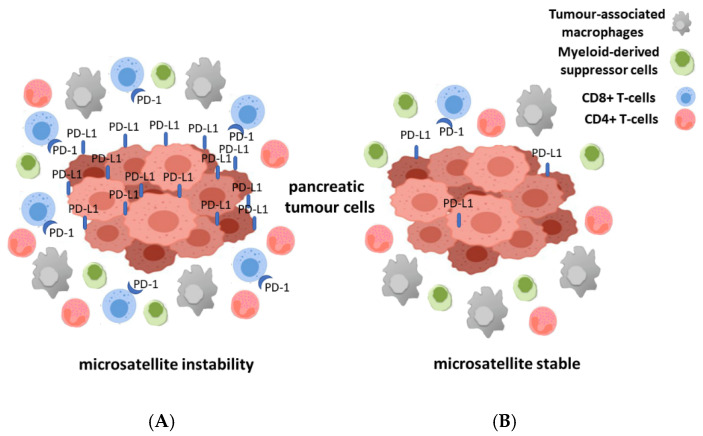
Illustration of the different immune milieu for microsatellite instable (MSI) (**A**) and microsatellite stabile (MSS) (**B**) pancreatic cancer. The different number of immune cells especially of CD8^+^ and CD4^+^ T-cells, myeloid-derived suppressor cells (MDSCs) and tumor-associated macrophages (TAMs) in the tumor microenvironment of the two groups of pancreatic cancer is shown.

For a long time, pancreatic cancers have been regarded as tumors that are able to evade a host immune system, but considering new studies this assessment has been modified. Following new observations, it is widely accepted now that immune cell infiltration is present in many cancers and it also might represent a prognostic tool in pancreatic cancer [82,83,84,85]. In this new view, pancreatic tumors are infiltrated by different subgroups of T-cells. High infiltration rate of CD4^+^ and CD8^+^ T-cells and low number of regulatory T-cells seems to be related to a better prognosis [86,87]. A recent study demonstrated that a high number of CD3^+^ and CD8^+^ T-cells are indicators of a more favorable prediction, moreover by combining them both into an immune cell score the prognostic value can be further improved [88]. It is known that in MSI pancreatic cancer the amount of CD8^+^ T-cells at the invasive front in addition to the expression level of PD-1 and PD-L1 is higher than in pancreatic cancer with intact mismatch repair system [30] (Figure 1). Nevertheless, the dense stromal tissue present in the tumor microenvironment might be the reason behind the wide variations in density of T-cells within the tumor area. Furthermore, compared to other tumors, pancreatic tumors are often characterized by a low level of activated cytotoxic CD8^+^ T-cells and an intense infiltration of immune-suppressive cells such as tumor-associated macrophages (TAMs), myeloid-derived suppressor cells (MDSCs), and regulatory CD4^+^ T-cells (Tregs) [85,89,90,91,92,93].

In contrast to colon cancer, there is currently no common consent regarding measuring immune response in pancreatic tumor [94]. A promising diagnostic advance in identifying MSI in pancreatic cancer patients has been done recently [95]. In this study, MSI was identified in liquid biopsies by testing circulating tumor DNA. This achievement overcomes the difficulties to obtain tumor tissue by means of traditional tissue biopsy from the pancreatic patients. Furthermore, in the same study, it was possible to monitor the effect of pembrolizumab targeted therapy by means of serial analysis of circulating tumor DNA. Recently, the advantage of using liquid biopsies for monitoring pancreatic cancer therapy has also been presented in a meta-analysis based on 19 studies including 1872 patients [96]. Therefore, analyzing circulating tumor DNA based on the non-invasive method of liquid biopsy could become a method to monitor the response to immune therapy in pancreatic cancer patients.

## 4. Chemotherapeutic Options in MSI Pancreatic Cancer Patients

Several studies with MSI colorectal cancer patients have shown that mismatch repair-deficient patients benefit differently from standard chemotherapy compared to microsatellite stable (MSS) ones, therefore 5-fluoruracil-based regimes for instance are not recommended for this colorectal cancer patient subgroup [97,98,99,100]. Additionally, pre-clinical and clinical studies provided evidence of elevated cytotoxic effects of some drugs in MSI tumors [101,102]. 

In general, mismatch repair deficiency alone is not a direct transforming factor for cells and only the subsequent accumulation of cancer specific further oncogenic mutations and other genomic alterations results in the develop of MSI tumors [103]. Moreover, on one hand, a deficient mismatch repair system might affect the malignancy through increased drug resistance (especially in case of methylating, alkylating, and platinum-containing agents). On the other hand, it results in a higher rate of potentially immunogenic neo-antigens and higher response rate to immune therapy [58,104,105]. Drug resistance mechanisms in mismatch repair deficient tumors can occur by an increased tolerance to DNA damage, reduced cell-cycle arrest ability and defective apoptotic signaling [105,106,107].

Therefore, in line with the above-described facts pancreatic cancer patients with MSI also react differently to chemotherapy, as documented by two different studies [74,108]. In one study, it was shown that pancreatic cancer patients react significantly differently towards an adjuvant chemotherapy with pyrimidine analogue. Patients with MSI have no survival advantage when treated with 5-fluoruracil or gemcitabine-based chemotherapy whereas patients with intact mismatch repair system have a 10-month-prolonged DFS [74]. In another study, metastatic pancreatic cancer patients with deficient mismatch repair system showed better outcome (median OS of 16.5 months) compared to patients with intact mismatch repair system (median OS of 11.1 months) while undergoing FOLFIRINOX treatment [108]. Both studies underline the benefit of a more personalized treatment for pancreatic cancer patients.

Nevertheless, in pancreatic tumors, as with in other gastrointestinal cancers (e.g., hepatocellular, gastric, renal cell, and esophageal cancer), the immune-suppressive tumor microenvironment and low immunogenicity of the tumor is a hurdle that must be overcome. Furthermore, pancreatic cancer has a low rate of somatic mutations and a minimal neo-epitope presentation [90,91,109]. Combination therapies to increase immune responsiveness seems to be one possibility to overcome this limitation. Results of early clinical studies using immune-checkpoint inhibition in pancreatic cancers have been disappointing so far [110].

Since 2017, the PD-1 (programmed cell death protein-1; CD279) inhibitor pembrolizumab is approved for treatment of mismatch repair-deficient cancers irrespective of the tumor site by the U.S. Food and Drug Administration [81,111]. In general, PD-L1 (programmed death-ligand 1; CD274) is expressed on the surface of cancer cells but only rarely expressed by normal tissues [112]. After binding of PD-1 to PD-L1 the proliferation of antigen-specific T-cells in lymph nodes is suppressed and apoptosis of Tregs is reduced. Therefore, antibodies targeting PD-1 and PD-L1 can restore anti-tumoral immunity by stimulating endogenous immune response [113]. 

Treatment based on pembrolizumab alone has been only successful in MSI-high (instability in at least two of the five microsatellites markers) pancreatic cancer patients but not in MSI-low (instability in only one of the five microsatellites markers) patients [114,115].

In some studies, anti-PD-1/PD-L1 molecules have been administered together with vaccine, conventional chemo- or radiotherapy for treatment of MSI-low pancreatic cancer with the aim to transform an immune-suppressive to an immune-active microenvironment [92,116,117,118,119]. 

Based on relevant in vivo experiments and a small clinical study with ten pancreatic cancer patients, it was concluded that chemotherapy with gemcitabine most probably has the capacity to enhance responses to immunotherapy [117,120]. Even if gemcitabine suppressed memory T-cells, it was able to increase naïve T-cell function [117]. In agreement with this observation, the combination therapy of gemcitabine with antibodies targeting PD-1 and PD-L1 induced a significant synergistic anti-tumor effect in mouse models of pancreatic tumor [118]. Phase I/II studies involving anti-PD-1 and PD-L1 in pancreatic cancer are summarized in Table 2.

17 patients were included in a phase I/II study and treated with gemcitabine, nab-paclitaxel, and pembrolizumab. The maximum tolerated dose of this treatment was pembrolizumab 2 mg/kg every 21 d, gemcitabine 1000 mg/m^2^ and nab-paclitaxel 125 mg/m^2^ on days 1 and 8 every 21 d. Among 11 evaluable patients, disease control rate (DCR) was 100%, median PFS was 9.1 months and OS 15 months [116]. In another phase I study the combination of nab-paclitaxel 125 mg/m^2^ and gemcitabine 1000 mg/m^2^ on days 1–8–15 were also tested with nivolumab at the dose of 3 mg/kg (days 1 and 15) on 50 patients. The combination was safe; however, activity beyond standard chemotherapy doublet was not registered, with a median PFS of 5.5 and median OS of 9.9 months [121]. In a study of second-line treatment for advanced pancreatic cancer after progression to 5-FU or gemcitabine-based regimens, 65 patients were randomized to monotherapy with durvalumab (1.5 g every 4 w) or the combination of durvalumab and tremelimumab (75 mg every 4 w). A partial response (PR) which persisted 9 months was observed in one patient (3.1%) in the combination arm and in 9.4% of patients’ disease control was reached. 6.1% of patients in the durvalumab alone arm demonstrated PR and disease control. Due to lack of efficacy signal demonstrated in the first part of the study, the trial was not further conducted to assess efficacy by overall response rate (ORR) [122]. 

In another attempt, a vaccine based on irradiated, allogenic pancreatic tumor cells expressing granulocyte-macrophage colony-stimulating factor (GVAX), was combined with PD-1L and PD-1 inhibitors. Combination of this vaccine with immune-checkpoint inhibitors was able to render pancreatic cancer accessible to immunotherapy [119]. In a phase II preliminary randomized study, all participants received two doses of low-dose cyclophosphamide to inhibit T-cells prior to GVAX vaccine activating a broad antigenic response (Cy/GVAX). Then, the patients were randomized between the prosecution of the Cy/GVAX protocol for six further cycles or switch to a different vaccine (CRS-207). CRS-207 is a vaccine of live-attenuated, mesothelin-expressing *Listeria monocytogenes*. Treatment with both vaccines was well-tolerated and combined treatment with CRS-207 and Cy/GVAX resulted in improved median OS of 6.1 months compared to 3.9 months for Cy/GVAX treatment alone [130]. The efficiency of these vaccines was analyzed further in a subsequent study (ECLIPSE study) based on 213 pancreatic cancer patients who received at least two prior treatment regimens. Patients were randomized and received either both vaccines (Cy/GVAX and CRS-207; arm A) together, or CRS207 alone (arm B), or single-agent chemotherapy according to physician’s choice (arm C). At the final analysis, there was no OS difference between the three treatment arms regarding median OS, with values of 3.7, 5.4 and 4.6 months, respectively. Thus, combining CRS-207 and Cy/GVAX vaccine resulted not in an improved survival over standard chemotherapy [131]. Another phase II study, the STELLAR study, included pancreatic cancer patients who received one prior treatment regimen. Ninety-three patients received first vaccination with Cy/GVAX followed by CRS-207 vaccine and were then randomized into two arms—either with or without nivolumab treatment. Every three weeks nivolumab was administered at a dose of 3 mg/kg for six total cycles together with Cy/GVAX followed by CRS-207. Median OS did not differ significantly between the two arms (5.9 months and 6.1 months, respectively) and the addition of nivolumab did not improve survival outcomes [123].

Vitamin D receptor agonist (paricalcitol) has demonstrated activity in sensitizing pancreatic cancer lesions to immune-checkpoint blockade by reducing the activity of MDSCs and Tregs [132]. In advanced chemotherapy-naïve pancreatic cancer patients, treatment with paricalcitol was combined with nivolumab, nab-paclitaxel, gemcitabine, and cisplatin. Preliminary results on ten treated patients reported partial regression in eight patients (80% of PR) and stable disease in two patients (100% of DCR). In this study, 8.2 months were indicated as median PFS and up to now no data for median OS are available [124].

MDSCs and TAMs account for the immunosuppressive microenvironment of pancreatic cancer [133]. TAMs and MDSCs are recruited to the tumor stroma by high levels of colony-stimulating factor 1 (CSF-1) secreted from pancreatic tumor cells [134]. Inhibition of the CSF-1 receptor resulted in reprogramming of TAM to M1 (more immunogenic cells), increased cytotoxic T-cell infiltration and reduced the amount of Tregs in the tumor microenvironment [135]. The CSF-1 receptor (CSF-1R) inhibitor cabiralizumab was administered together with nivolumab in a phase I study for pretreated advanced pancreatic cancer patients. Four out of 31 patients had an ORR of 13% and of special interest all belong to the MSS subgroup [136]. Another phase I/II trial tested the combination of lacnotuzumab (anti-CSF-1 monoclonal antibody) with spartalizumab (PD-1 inhibitor). From the 13 patients with advanced pancreatic cancer, six patients had disease control with this combination and three of six had a disease control superior to 300 d [136,137].

PD-1 inhibitors have been demonstrated to execute also synergistic effects with focal adhesion kinase (FAK) inhibitors. FAK is often overexpressed in pancreatic cancer and has well known oncogenic properties [136]. The FAK inhibitor defactinib was combined with pembrolizumab and gemcitabine in the frame of a phase I study. Among ten evaluable pancreatic cancer patients in maintenance treatment after chemotherapy with gemcitabine and nab-paclitaxel, 60% had stable disease and 10% progressed. 4.6 months was the median time on treatment. In the refractory cohort, included patients progressed to first-line chemotherapy, 50% had stable disease. Median progression-free disease was 2.9 months and median OS was 7.6 months. In the dose escalation cohort, one more patient progressed. Of interest, both progressing patients have been identified as MSS [127]. 

Ibrutinib, a bruton tyrosine kinase (BTK) inhibitor, plays a role in the immunomodulation of pancreatic cancer tumor microenvironment [138] and therefore the BTK-inhibitor ibrutinib was tested together with nab-paclitaxel and gemcitabine in advanced pancreatic cancer patients as first-line treatment (Resolve study) [139]. Furthermore, in a phase I/II study ibrutinib was also combined with PDL-1 inhibitor durvalumab but OS was poor with a median survival of 4 months only [128]. 

Another treatment strategy aimed at activating T-cells. One example is the use of AM0010, a pegylated IL-10. AM0010 was tested in second-line treatment of advanced pancreatic cancer in combination with FOLFOX (folinic acid, 5-fluorouracil (5-FU), oxaliplatin) chemotherapy. In this study, 25 participants were treated with AM0010 (5 ug/kg per day) in combination with FOLFOX (every 14 d). ORR was 15.8% with a DCR of 78.9%. Median PFS was 3.5 months, median OS was 10.2 months and 1-year survival 43% [140]. The subsequent SEQUOIA trial (phase III) tested the combination of pegylated IL-10 (pegilodecakin) together with FOLFOX in gemcitabine refractory pancreatic cancer patients. The control arm received only chemotherapy with FOLFOX. Surprisingly, the addition of pegilodecakin to FOLFOX did not improve efficacy (PFS, OR, ORR) [141]. 

A different target is CD73, a cell surface enzyme often up-regulated in pancreatic cancer. CD73 exerts an immunosuppressive effect by generating extracellular adenosine [142]. Oleclumab, a human monoclonal antibody that binds to CD73, was tested in combination with durvalumab. As expected, production of immunosuppressive adenosine was reduced and the amount of CD8^+^ T-cells in the tumor microenvironment increased after oleclumab treatment. Partial regression was observed in 10% of cases, while DCR was present in 25% of patients [129]. Another enzyme with immunosuppressive effect is indoleamine-2,3-dioxygenase-1 (IDO1) [143]. Increased IDO-1 expression on tumor cells results in NK-cell and T-cell suppression, Tregs activation and promotion of immune tolerance [144]. In a phase II study, 135 patients were evaluated for first-line treatment with nab-paclitaxel, gemcitabine, and the IDO1 inhibitor indoximod. In this study, the reported median OS was 10.2 months and ORR was 46.2%, with 45.2% of partial repression. The intratumoral CD8^+^ T-cell density was higher in responders of this treatment than in non-responders [145].

## 5. Ongoing Studies

Currently, several clinical studies (NCT02648282, NCT03336216, NCT03190265, NCT02451982, NCT02558894, NCT02309177, NCT02303990, NCT02546531, NCT01714739, NCT03404960, NCT03006302) are ongoing based on combination of a vaccine (GVAX or CRS-207) and anti-PD-1 therapy in pancreatic cancer patients. Of special interest is a study in which PD-1 inhibitor nivolumab is combined with cabiralizumab, an antibody that targets TAMs (NCT02526017). In this study, the metastatic pancreatic cancer patients with MSI are responding well to the combination therapy [146]. 

Anti-tumor cell activity can also be activated by using CD40 agonists [147]. They can bind to CD40 receptor which is present on B-cells, antigen-presenting cells as well as on activated CD4^+^- and CD8^+^ T-cells. CD40 agonist most probably act in a T-cell depending manner as well as by reprogramming macrophages to TH1 cells [148]. For treatment of pancreatic cancer, a combination of CD40 agonist with radiotherapy is currently under investigation in one clinical study (NCT02311361) and combination of CD40 agonist with chemotherapy based on gemcitabine, nab-paclitaxel and nivolumab is used in a clinical phase I/II trial (NCT 03214250). According to the preliminary results of a phase II study (COMBAT study) CD40 agonists can increase the survival of metastatic pancreatic cancer patients. The number of TREG (regulatory T-cells) cells are diminished by 50% whereas CD3^+^-cells and CD8^+^-T-cells increased 15-fold and 2-fold, respectively, in liver biopsies [149].

HDAC inhibition is known to result in an increased MHC (major histocompatibility complex) class I presentation and decreased PD-L1 expression [150]. Therefore, entinostat (HDAC I and III inhibitor) is currently used in combination with the checkpoint inhibitor nivolumab for evaluation of possible synergistic effects in the frame of a phase II study (NCT03250273) for treating pancreatic cancer and advanced cholangiocarcinoma patients. It will be of interest to see the results of this combination study.

Another attempt to overcome the immune-suppressive microenvironment of pancreatic cancer is based on a combination of Janus kinase- and phosphoinositide-3-kinase-inhibitors with radiotherapy [151]. Up to now, this combination has been limited to pre-clinical studies and has not been evaluated further in clinical trials.

## 6. Conclusions and Perspectives

Interest in mismatch repair-deficient pancreatic cancer is growing, since it is becoming more evident that also in pancreatic cancer immune-checkpoint inhibition therapy is efficient and provides a potential good response in this subgroup of patients. However, the available data about mismatch repair-deficient pancreatic cancer is still limited and sometimes controversial. This is mainly related to different detection methods and sample sizes. Therefore, it is of pivotal importance to standardize detection tools for deficiency in mismatch repair system, establish screening programs, and select pancreatic cancer patients that will benefit from immune therapies. Next-generation sequencing (NGS) could help to overcome these issues [38]. Determination of the MSI status by NGS is possible with a commercially available assay [152] so that variations between laboratories are minimized. Another advantage of this assay for MSI evaluation is that it does not require matched samples from normal tissue. Furthermore, NGS-based methods cover a broader range of microsatellite loci; thus, it is not limited to the five microsatellite sites used in the PCR-based method [152]. Nevertheless, the high investment costs and the longer time needed to perform NGS run and bioinformatic analysis in comparison to PCR and immunohistochemistry-based MSI analysis methods are the costs for a more accurate diagnosis.

Most probably, this step toward personalized medicine might increase in the future the survival of this subgroup of patients.

## Figures and Tables

**Table 1:** Major studies assessing MSI in pancreatic cancer.

Author/Year	Study Population	Methodology	MSI in %
Luipinacci/2018 [30]	445	IHC on resected samples from consecutive patients at multiple centers	1.6
Hu/2018 [31]	833	NGS, PCR-based and IHC on resected samples from consecutive patients	0.8
Liu/2014 [49]	36	IHC on resected and metastatic selected patients with acinar cell carcinoma	13.8
Abe/1996 [52]	44	PCR based on resected samples	15.9
Yamamoto/2001 [53]	103	PCR-based and IHC on resected samples from partially selected patients (3 Lynch Syndrome patients added to a series of 100 patients from multiple centers)	15.5
Abraham/2002 [54]	21	PCR based on resected samples (17 patients) and core biopsies (4 patients) from selected patients with acinar cell carcinoma	7.7
Tomaszewska/2003 [55]	30	IHC on resected samples from consecutive patients	0.0
Luttges/2003 [56]	23	PCR-based and IHC on resected samples from selected patients (extensive invasive mucinous component)	4.3
Maple/2005 [57]	35	PCR-based and IHC on selected patients (long-term survivors; ≥3 years)	8.6
Nakata/2002 [58]	46	PCR based on resected samples from consecutive patients	17.4
Fujii/2009 [59]	21	PCR based on resected samples	0.0
Laghi/2012 [60]	338	PCR-based and IHC on samples from consecutive patients at multiple centers	0.3
Han/1993 [61]	9	PCR based on resected samples	67.0
Seymour/1994 [62]	33	PCR based on resected samples	21.2
Brentnall/1995 [63]	17	PCR based on pancreatic juice	75.0
Venkatasubbarao/1998 [64]	14	PCR based on resected samples	57.0
Ouyang/1997 [65]	51	PCR based on resected samples	14.0
Ouyang/1998 [66]	60	PCR based on resected samples	15.0
Goggins/1998 [67]	82	PCR based on samples from consecutive patients	3.7
Ghimenti/1999 [68]	21	PCR based on samples from consecutive patients	67.0
Wilentz/2000 [69]	18	IHC and PCR based on samples from selected patients with medullary histology	22.2
Ueki/2000 [70]	36	PCR based on resected samples	11.1
Nakata/2003 [71]	55	IHC on resected samples	9.2
Ottenhof/2012 [72]	78	IHC on resected samples from patients at multiple centers	12.8
Mitsuhaski/2015 [73]	283	Methodology not specified	0.0
Riazy/2015 [74]	265	IHC on resected samples from consecutive patients	15.4
Grant/2015 [75]	38	NGS on blood samples from patients	2.6
Connor/2017 [76]	255	NGS, PCR-based and IHC on resected samples (243 primary tumors and 12 metastases)	1.7
Lucchini/2020 [78]	8323	Systematic review of 34 studies	2.0

Legend: IHC: immunohistochemistry; NGS: next-generation sequencing; PCR: polymerase chain reaction.

**Table 2:** Phase I/II studies involving anti-PD-1 and PD-L1 in metastatic pancreatic cancer.

Author/Year	Study Phase	N° pts	N° MSI-H/PD-L1-H (%)	Anti-PD-1/PD-L1 Agent	Combination Agent	Outcomes
Weiss/2018 [116]	I/II	17	9 (53) MSI-H	pembro	gemcitabine/nab-paclitaxel	DCR 100%, mPFS 9.1 mo, mOS 15 mo
Wainberg/2020 [121]	I	50	12 (24) PD-L1 ≥ 1	nivo	gemcitabine/nab-paclitaxel	mPFS 5.5 mo, mOS 9.9 mo
O’Reilly/2019 [122]	II	65	8 (12) PD-L1 ≥ 25	durva	tremelimumab	DCR 9.4%, PR 3.1%
Tsujikawa/2020 [123]	II	93	NA	nivo	Cy/GVAX/CRS-207	mOS 5.9 mo
Borazanci/2018 [124]	II	25	NA	nivo	paricalcitol, gemcitabine/nab-paclitaxel/cisplatin	PR 80%, DCR 100%, mPFS 8.2 mo, mOS NR
Wainberg/2017 [125]	I	31	NA	nivo	cabiralizumab	ORR 13% in MSS
Calvo/2018 [126]	I/II	50	NA	sparta	lacnotuzumab	DCR 46%
Wang-Gillam/2020 [127]	I	20	NA	pembro	defactinib/ gemcitabine	mPFS 2.9 mo, mOS 7.6 mo
Hong/2019 [128]	I/II	49	NA	durva	ibrutinib	mOS 4 mo
Overman/2018 [129]	I	20	NA	durva	oleclumab	PR 10%, DCR 25%

Legend: CRS-207: vaccine; Cy: cyclophosphamide; DCR: disease control rate; durva: durvalumab; GVAX: irradiated allogenic pancreatic tumor cells vaccine; mo: months; mOS: median overall survival; MSI-H: microsatellite high; mPFS: median progression-free survival; MSS: microsatellite stable; N°: number; NA: not available; nivo: nivolumab; NR: not reached; ORR: overall response rate; PD-L1-H: PD-L1 high; pembro: pembrolizumab; PR: partial response; sparta: spartalizumab.
